# Distinct roles for histone chaperones in the deposition of Htz1 in chromatin

**DOI:** 10.1042/BSR20140092

**Published:** 2014-09-19

**Authors:** Hongde Liu, Min Zhu, Yawen Mu, Lingjie Liu, Guanghui Li, Yakun Wan

**Affiliations:** *State Key Laboratory of Bioelectronics, Southeast University, Nanjing 210096, China; †The Key Laboratory of Developmental Genes and Human Disease, Ministry of Education, Institute of Life Sciences, Southeast University, Nanjing 210096, China; ‡Jiangsu Nanobody Engineering and Research Center, Nantong 226010, China

**Keywords:** histone chaperone, Htz1 deposition, nucleosome structure, ChIP-chip, chromatin immunoprecipitation and tiling microarray chips, GO, gene ontology, HA, haemagglutinin, HAT, histone acetyltransferase, Nap1, nucleosome assembly protein 1, NFR or NDR, nucleosome-free (depleted) region, PCC, Pearson correlation coefficient, Pol II, polymerase II, TSS, transcription start sites, TTS, transcription termination site

## Abstract

Histone variant Htz1 substitution for H2A plays important roles in diverse DNA transactions. Histone chaperones Chz1 and Nap1 (nucleosome assembly protein 1) are important for the deposition Htz1 into nucleosomes. In literatures, it was suggested that Chz1 is a Htz1–H2B-specific chaperone, and it is relatively unstructured in solution but it becomes structured in complex with the Htz1–H2B histone dimer. Nap1 (nucleosome assembly protein 1) can bind (H3–H4)_2_ tetramers, H2A–H2B dimers and Htz1–H2B dimers. Nap1 can bind H2A–H2B dimer in the cytoplasm and shuttles the dimer into the nucleus. Moreover, Nap1 functions in nucleosome assembly by competitively interacting with non-nucleosomal histone–DNA. However, the exact roles of these chaperones in assembling Htz1-containing nucleosome remain largely unknown. In this paper, we revealed that Chz1 does not show a physical interaction with chromatin. In contrast, Nap1 binds exactly at the genomic DNA that contains Htz1. Nap1 and Htz1 show a preferential interaction with AG-rich DNA sequences. Deletion of *chz1* results in a significantly decreased binding of Htz1 in chromatin, whereas deletion of *nap1* dramatically increases the association of Htz1 with chromatin. Furthermore, genome-wide nucleosome-mapping analysis revealed that nucleosome occupancy for Htz1p-bound genes decreases upon deleting *htz1* or *chz1*, suggesting that Htz1 is required for nucleosome structure at the specific genome loci. All together, these results define the distinct roles for histone chaperones Chz1 and Nap1 to regulate Htz1 incorporation into chromatin.

## INTRODUCTION

The histone variant H2A.Z, or Htz1 in *Saccharomyces cerevisiae*, replaces H2A in nucleosomes and has diverse roles in eukaryotic cells [1]. Histone H2A.Z regulates transcription and it is partially redundant with nucleosome remodelling complexes [1]. During transcription initiation, H2A.Z establishes open chromatin near TSSs (transcription start sites) Genome-wide studies have revealed that two H2A.Z-containing nucleosomes flank an NFR or NDR [nucleosome-free (depleted) region] near the TSSs [2–4] and Htz1 proximity to the TSS influences gene expression [5]. H2A.Z is enriched in both centromeres and telomeres [6–8], and a lack of Htz1 can repress telomere-proximal genes [9] and abolish cell-cycle progression in yeast [10]. The molecular mechanisms for these phenomena are not very clear. It is known that H2A.Z is lost at promoters and is enriched at the centromere when mitosis begins in mouse trophoblast stem cells [11]. Furthermore, in differentiating murine ESCs (embryonic stem cells), Htz1 deposition increases chromatin accessibility [12]. H2A.Z also has immunology-related functions, showing a specific distribution pattern around the genome variation sites in human CD4^+^ T cells [13].

In *S. cerevisiae*, because of the importance of Htz1, the molecular mechanism for incorporating Htz1 into nucleosomes is of critical interest. Depositing and evicting Htz1 from chromatin requires the coordination of the SWR1 remodelling complex, specific histone modifications and histone chaperones. Zhang et al. showed that Htz1 deposition partially relies on Gcn5, an HAT (histone acetyltransferase), and Bdf1, an SWR1 complex member that binds acetylated histones [3]. In a human cell line, purified Htz1-interacting complexes contained the components of the SRCAP chromatin-remodelling and TIP60 HAT complexes [14]. Additionally, the acetylation of H3K56Ac (histone H3 at lysine 56) alters the substrate specificity of SWR1 in exchanging H2A with Htz1 [15]. Htz1 incorporation is catalysed by the SWR1 complex in an ATP-dependent manner [16]. Importantly, both H2A-containing nucleosomes and Htz1–H2B dimers specifically stimulate the ATPase activity of SWR1 [17].

Chz1 is an important component of SWR1-dependent Htz1 replacement. It is a Htz1–H2B-specific chaperone that delivers Htz1 for H2A substitution [18]. It is relatively unstructured in solution, but it becomes structured in complex with the Htz1–H2B histone dimer [19]. Two short alpha-helices and an extended linker roughly follow the Htz1 shape (PDB ID: 2JSS). The chaperone–histone Chz1–Htz1–H2B complex is mainly driven by attractive electrostatic interactions [20]. Our previous work suggested that Chz1 regulates H2B ubiquitination and subtelomeric anti-silencing which is independent of Htz1 [21]. Chz1 can facilitate the disfavoring property of Spt16 to Htz1-containing genes [22].

Nap1 (nucleosome assembly protein 1) is also an important component for Htz1 replacement of H2A. Nap1 can bind (H3–H4)_2_ tetramers, H2A–H2B dimers and Htz1–H2B dimers [9,16,23]. Nap1 binds H2A–H2B dimers in the cytoplasm and the complex shuttles into the nucleus [24–26]. Moreover, Nap1 functions in nucleosome assembly by competitively interacting with non-nucleosomal histone–DNA. Additionally, unincorporated Htz1–H2B dimers form a complex with Nap1 [26,27]. These multiple roles make Nap1 very important for normal cell activity. In yeast, deleting the *nap1* gene significantly increases atypical histone–DNA complexes [26]. Xue et al. suggested that Nap1 genetically interacts with Pol II (polymerase II) and regulates H3K9ac [28]. Histone chaperones are known to escort histone variants through complicated mechanisms [29]. Specifically, Chz1 does not appear to interact with Htz1 in the cytoplasm, rather Nap1 interactions with Htz1–H2B dimers maintain a soluble pool of Htz1 for transport to the nucleus where Chz1–Htz1 interactions can occur [30].

Despite the advancements mentioned above, the roles of Chz1 and Nap1 in the deposition and eviction of Htz1 remain largely unknown. Two important open questions are: (1) do both Chz1 and Nap1 escort Htz1 from the cytoplasm to chromatin; and (2) what roles do Chz1 and Nap1 have in depositing and assembling Htz1-containing nucleosomes. In this study, we used ChIP-chip (chromatin immunoprecipitation and tiling microarray chips) to determine the genome-wide binding loci of Htz1, Chz1 and Nap1, in both wild-type and chaperone-deletion strains of *S. cerevisiae*. We combined these binding location data, with new nucleosome maps built by next generation sequencing. Our data demonstrate distinct roles for Chz1 and Nap1 in incorporating Htz1 into chromatin. Chz1 does not bind to genomic DNA, whereas Nap1 and Htz1 competitively bind genomic DNA. Deleting the *chz1* gene decreased Htz1 binding. In contrast, deleting the *NAP1* gene increased Htz1 binding. Interestingly, the acetylation level of Htz1 is dramatically increased in *chz1* mutant. The nucleosome occupancy of Htz1-bound genes decreased in *htz1* or *chz1* mutants. Our results elucidated the functional roles of Nap1 and Chz1 in depositing Htz1 on chromatin.

## MATERIALS AND METHODS

### Yeast strains and growth conditions

The yeast strains that were used in this study are indicated in Supplementary Table S1. The genomic integration of genes encoding C-terminal HA (haemagglutinin) fusions was performed using a PCR-based, one-step method for gene modification [31]. Yeast transformations were performed using a lithium acetate/polyethylene glycol-based method [32]. The strains were verified by PCR analysis of the tagged gene loci and Western blot analysis of the fusion proteins. The strains were cultured at 30°C in YPD medium [1% (w/v) yeast extract/2% (w/v) peptone/2% (w/v) glucose] unless otherwise indicated.

### Chromatin fractionation assay

The chromatin fractionation assay was performed as [33] with minor modifications. Briefly, after spheroplasting, cells were washed with the washing buffer. The pellet of spheroplasts was then resuspended in EB buffer. The cells were incubated on ice for 5 min for lysis with vortexing every 1 min to make the total cell lysate. Then 100 μl of cell lysate was placed on the top of 200 μl of EBX-S buffer. The mixture was spun at 12 000 rpm for 10 min at 4°C. There was a while chromatin pellet, a clear sucrose layer, and a yellow supernatant fraction on top. Ten microliters of the upper yellowish liquid was removed and kept as the supernatant fraction. The white chromatin pellet was washed and resuspended in the EBX buffer, and the SDS sample buffer was added to the protein sample.

### Western blot

Proteins samples were separated by SDS–PAGE and transferred to nitrocellulose membranes. The nitrocellulose membranes were blocked with 5% (w/v) non-fat dried skimmed milk powder in PBST solution. HA-tagged proteins were detected with a 1:1000 dilution of mouse monoclonal anti-HA antibody. Myc-tagged proteins were detected with a 1:1000 dilution of mouse monoclonal anti-Myc antibody. Rabbit polyclonal antibodies were used to detect PGK1 (1:5000 dilution), Htz1 (1:1000 dilution), Htz1K14 acetylation (1:1000 dilution) and Chz1 (1:2000 dilution).

### RNA preparation and gene expression profile

Yeast cultures were grown at 30°C to a density of 1×10^7^ cells/ml. The cells were collected and immediately frozen in liquid nitrogen. Total RNA was isolated by hot acid phenol extraction. The total RNA was treated with RNase-free DNase I and purified with a Qiagen RNeasy kit. Microarray labelling and hybridization reactions were performed [34]. Two-colour microarrays were performed using Agilent whole-genome *S. cerevisiae* arrays. All experiments were performed with duplicate experimental and technical replicates of each condition. Genes with a ≥ 2-fold change in expression compared with the expression in the relevant wild-type strains were considered to be significantly affected.

### Chromatin immunoprecipitation

For each chromatin immunoprecipitation experiment, yeast strains were grown in the YPD medium to an OD_600_ of 1.0. ChIP was performed as described previously [35]. In brief, proteins were cross-linked to their respective DNA-binding sites with 1% (v/v) formaldehyde for 1 h at room temperature. Chromatin was then disrupted by glass bead lysis and sheared to an average size of 400 bp. Sheared chromatin lysates were incubated with antibody-conjugated magnetic beads overnight at 4°C with rotation. Following incubation, crosslinks were reversed in both the ChIP and whole-cell lysate fractions, and samples were analysed by DNA tiling microarrays.

### ChIP-chip

For genome-wide binding analysis of HA–Htz1 and Nap1-9xMyc, ChIP was performed as described above. Linkers were annealed to the ends of the ChIP and input [WCE (whole cells extract)] DNA samples, and DNA was then amplified by PCR. Amplified DNA from the IP and input samples were labelled using a ULS aRNA Fluorescent Labelling DNA Kit (Kreatech). Labelled DNA from the ChIP and input samples were hybridized to *S. cerevisiae* whole-genome tiling arrays (4×44 k; Agilent). Data were extracted using Agilent Feature Extraction software and analysed with Agilent ChIP Analytics software (Agilent).

### Nucleosome-positioning analysis

Wild-type, *htz1*Δ, *chz1*Δ and *nap1*Δ cells were grown in the YPD medium to an OD_600_ of 1.0. All cells were then treated with 1% (v/v) formaldehyde for 20 min and incubated with 125 mM glycine for 5 min. Cell permeabilization, micrococcal nuclease digestion, protein degradation and DNA purification steps were performed as previously described [33]. DNA samples were treated with RNase A and were separated in a 2% (w/v) agarose gel to assess the nucleosomal content. Bands corresponding to mononucleosomal DNA were extracted using a Qiagen gel extraction kit (Qiagen). Mononucleosomal DNA libraries were prepared and sequenced using an Illumina Genome Analyzer II (Illumina Inc.) according to the manufacturer's instructions.

### Coordinates of TSSs, TTSs (transcription termination sites) and REG sites

The genomic coordinates of TSSs and TTSs were retrieved from the Tables function of the University of California, Santa Cruz (UCSC) Genome Browser (http://genome.ucsc.edu) [36]. The experimentally identified DNA regulatory regions, transcription factor-binding sites, and regulatory variants were also retrieved from UCSC [37]; these data are called REG sites. The transcription rate data of yeast genes were retrieved from a previous report [38]. The data of TATA-box containing and TATA-less genes were previously published [39].

### Binding profiles of Htz1 and Nap1

Binding profiles for the whole genome were generated using the binding log_2_ ratio signal from ChIP-chip experiments. The binding of any genomic region that is covered by a probe is represented by the log_2_ binding ratio of the probe; the binding of a region that is not covered by a probe is zero. An Htz1-bound gene has at least one Htz1-bound probe that is identified within the region from 500-bp upstream to 300-bp downstream of the TSS. Htz1-bound probes are identified by two criteria: the central probe's binding ratio is >2 and the *P* value is <0.05, and the *P* value of either of the two neighbouring probes is <0.25. The Nap1-bound genes were identified similarly. The numbers of Htz1-bound and Nap1-bound sites are shown in Supplementary Figure S1.

### Occupancy patterns of Htz1 and Nap1

The occupancy pattern of Htz1 (or Nap1) around special sites (such as TSSs, REG sites and TTSs) is represented with average binding ratio (log_2_) by aligning the binding profile for each gene at the special site.

### GO (gene ontology) analysis

To analyse the dynamics of Htz1 binding, 5419 yeast genes were grouped into 40 gene function clusters according to their GO annotation using GO Slim Mapper (http://www.yeastgenome.org/cgi-bin/GO/goSlimMapper.pl). Then, the number of Htz1p-bound genes was counted in each cluster in the wild-type and deletion mutant strains, and the *P* value of the enrichment was calculated with a hypergeometric distribution. To compare the binding dynamics, the PCC (Pearson correlation coefficients) of the average binding ratio (log_2_) profile for each gene cluster were calculated in the wild-type and deletion mutant strains. Additionally, we directly performed a GO enrichment analysis for Htz1-bound genes using GO Term Finder (http://www.yeastgenome.org/cgi-bin/GO/goTermFinder.pl).

### Identification of motifs

DNA sequences of 60 bp around the centre sites of the bound probes were extracted and submitted to MEME to generate the motifs using the default settings of MEME [40].

### Nucleosome occupancy data

The raw sequencing reads were mapped on the *S. cerevisiae* strain S288c genome using Illumina CASAVA software with default ELAND alignment parameters. Only the uniquely mapped reads with less than 1 bp mismatch were used in further analysis. The nucleosome occupancy was obtained as follows. First, the length of each read was extended 73 bp in the 3′ direction. Then, the Waston-strand reads and Crick-strand reads were oppositely shifted 73 bp. Finally, the occupancy of each genomic site was calculated as the sequencing depth. The final nucleosome occupation is represented as the fold of the occupancy relative to the average occupation.

### Identification of nucleosome dyad sites

The dyad coordinates of nucleosomes were determined with a wavelet transformed-based algorithm. The nucleosome occupancy signal was first smoothed with Daubechies wavelet at level 4. Then, the peaks that satisfied the following two criteria were identified: (1) the height is >1.2 (the occupation is 1.2-fold higher than the average occupation); and (2) the FWHH (full-width at half-height) is not <73 bp.

### Nucleosome occupancy patterns

The nucleosome occupancy patterns near the special sites (TSSs, REG sites and TTSs) were represented by the average nucleosome occupancy profile, which was calculated by summing the occupancy signal at each genomic site and then dividing the summated signal by the gene number [41].

## RESULTS

### Genome-wide localizations of the histone variant Htz1 and its chaperones Chz1 and Nap1

#### Chz1 does not physically interact with chromatin

**Figure 1:**
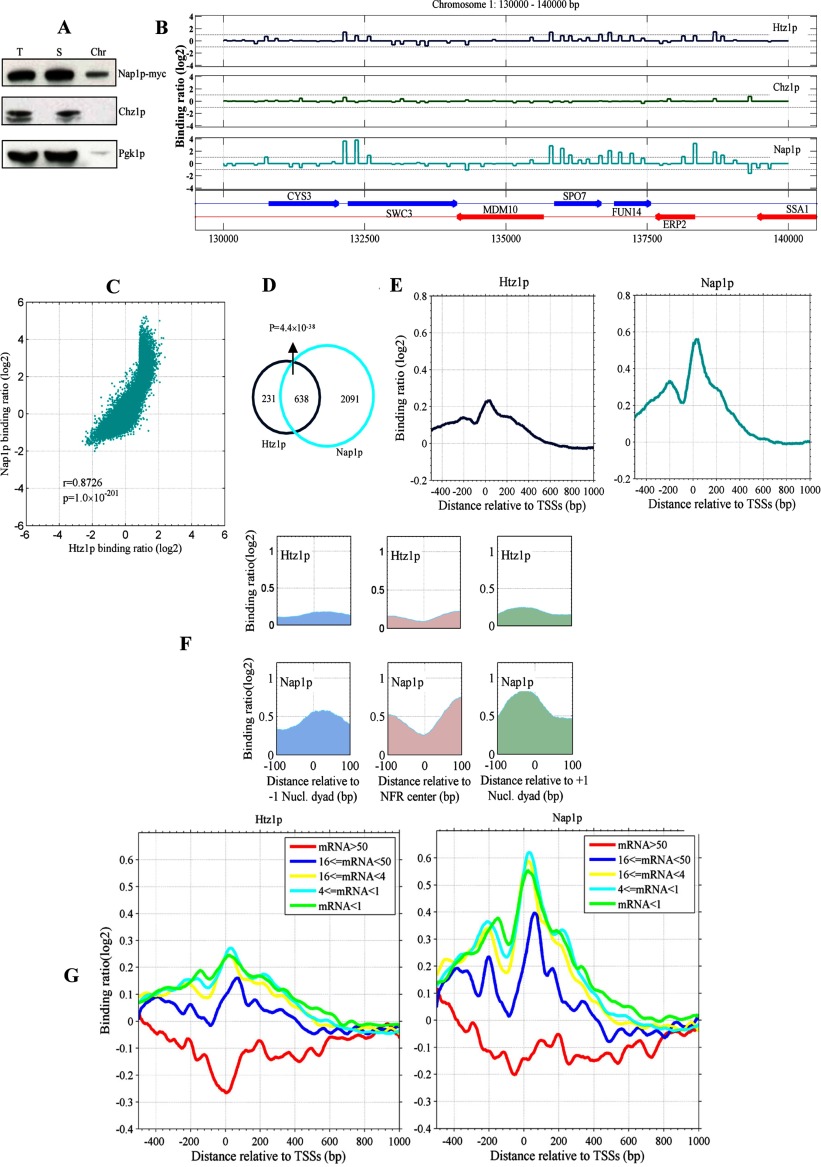
Binding of Htz1, Chz1 and Nap1 on the yeast chromosomes by Southern blot hybridization and ChIP-chip experiments (**A**) Chz1 does not physically interact with the chromatin. (**B**) The binding ratio (log_2_ scale) profiles of Htz1, Chz1 and Nap1 in the region of 130–140 kb on chromosome 1. The binding ratio refers to the ratio of the hybridization values for Cy3 [protein (Htz1 or Nap1)-bound DNA] versus Cy5 (genomic DNA). In the bottom panel, the arrows indicate the transcribed regions, the arrow direction indicates the direction of transcription, and the labels on the arrow indicate the gene names. Htz1 and Nap1 show a high binding signal, especially near the transcription start locus, indicating their binding on the chromatin, while the Chz1 signal is lower than log_2_(2). (**C**) Htz1 binding is highly correlated with Nap1 binding [*r*=0.8726, *P* value=1.0×10^−201^ (*t* test)]. (**D**) Venn diagram shows the numbers of Htz1-bound genes and Nap1-bound genes (see the Experimental procedures); the hypergeometric distribution *P* value is indicated for the overlapped gene number. (**E**) Binding patterns for Htz1 and Nap1 in the vicinity of TSSs. The average binding ratio profiles surrounding TSSs for 5419 genes of yeast are shown. The *x*-axis represents the distance to the TSSs; the *y*-axis represents the average probe binding ratio. Htz1 exhibits a trough flanked by two peaks on either side; Nap1 binding is similar to that of Htz1, but it has a higher signal level, especially around TSSs. (**F**) Occupancies of both Htz1 and Nap1 overlap nucleosome occupancies. The average binding ratio profiles relative to the −1 and +1 nucleosome dyad sites (left panel and right panel) and the centre of NFR (middle panel) are shown; the top panels indicate Htz1 binding, and the bottom panels indicate Nap1 binding. (**G**) The average binding ratio profiles of Htz1 (left) and Nap1 (right) for five gene groups with different transcription frequency (transcripts/hour). The transcription frequency data of yeast genes were retrieved from a previous report [26,53]. Correlation coefficient between the transcription frequency and the average binding ratio (−200 to 200 bp relative to TSS) is *r*=−0.93 (Fisher transformation, *P* value=1.0×10^−2^) for Htz1 and *r*=−0.97 (*P* value=1.0×10^−2.9^) for Nap1.

To resolve the roles of Chz1 and Nap1 in depositing Htz1 onto chromatin, we first used a chromatin fractionation assay to separate cells into the soluble protein fraction and insoluble, chromatin-bound fraction. We found that Nap1 directly associated with chromatin and showed a high affinity for chromatin. However, we did not detect a Chz1 signal in chromatin pellets (Figure 1A). Because Chz1 is a Htz1–H2B-specific histone chaperone [18], we infer that Chz1 does not physically interact with genomic DNA for facilitating to deliver Htz1–H2B to chromatin.

We also performed genome-wide ChIP-chip analysis of Htz1, Chz1 and Nap1. For each of the three proteins, we obtained the intensity values [binding ratio (log_2_) and *P* value] of more than 40000 probes that covered the entire yeast genome and constructed binding profiles for three proteins (Supplementary Figure S1). Our results indicated that Chz1 did not bind chromatin (log_2_ binding ratio<1) (Figures S1 and 1B), which was consistent with the chromatin fractionation test shown in Figure 1(A). In contrast, Htz1 and Nap1 had relatively high binding ratios (Figures S1 and 1B) particularly around TSSs (Figure 1B).

#### Nap1 overlays with Htz1 in binding to chromatin

The binding profiles of Htz1 and Nap1 were strongly correlated (*r*=0.8726; *P* value<10^−200^) (Figure 1C), suggesting that the occupancy of Nap1 significantly overlaps with that of Htz1. We defined a gene as Htz1-bound (or Nap1-bound) if there was at least one Htz1-bound probe (or Nap1-bound probe) in the region from 500 bp upstream to 300 bp downstream of the TSS (see the Experimental procedures section). Based on this criteria, 638 of the 839 targets for Htz1 were shared with Nap1 (*P* value=4.4×10^−38^) (Figure 1D), suggesting a significant overlap in chromatin binding between Htz1 and Nap1. However, we cannot exclude the possibility that the different binding of Htz1 and Nap1 in this study may be due in part to the different tags used in the ChIP-chip experiments (e.g. 9xMyc for Nap1 and HA for Htz1).

#### Nap1 is enriched at the nucleosomes near TSSs

To understand the organizations of Htz1 and Nap1 at promoters, we calculated the average binding ratio profiles for Htz1 and Nap1 by aligning the binding profiles for 5419 yeast genes at TSSs (Figure 1E). The profiles for both Htz1 and Nap1 showed a low density at ~80 bp upstream of TSSs, corresponding to the NFR, and peaks at the two immediate sides of the trough that decrease to the background level (i.e. 0) at about 600 bp downstream of TSSs (Figure 1E). Thus, Htz1 and Nap1 have similar distributions showing both factors enriched at promoter regions.

#### Htz1 occupancy overlaps with the nucleosome occupancy, and Nap1 binds the genomic DNA that wraps around Htz1

The intimate association between nucleosomes and Htz1 led us to explore the relative position of Htz1 to nucleosome dyads. We determined the nucleosome positions by the next generation sequencing and identified the position of nucleosome dyads using a wavelet-based algorithm. In the first (i.e. −1) nucleosome upstream of the TSS, Htz1 is ~+24 bp upstream of the dyad position; in the first (i.e. +1) nucleosome downstream of the TSS, Htz1 is ~−37 bp downstream of the dyad position (Figure 1F). At the centre of the NFR, the Htz1 profile shows a low trough (Figure 1F). Moreover, the position of Nap1 is very similar to that of Htz1 (Figure 1F). This suggests that the Htz1 occupancy overlaps with the nucleosome occupancy. Since our ChIP-chip experiments were performed with sonicated genomic DNA, we cannot infer that all chromatin associated Htz1 or Nap1 are in nucleosomes. Especially, the previous study has showed that Nap1 binding may inhibit non-nucleosomal binding of H2A–H2B to genomic DNA [26]. In conclusion, our data demonstrated that histone chaperone Nap1 can associate with genomic DNA wrapped with Htz1. We also noticed that the binding loci of Htz1 relative to nucleosome dyad sites were different in the −1 and +1 nucleosomes (Wilcox signed rank test, *P* value=1.4×10^−20^) (Figure 1F). This was probably caused by the different rotational conformation of the two nucleosomes (Figure 1F).

#### Htz1p and Nap1p are negatively correlated with transcription frequency

The enrichment of Htz1 near TSSs is closely correlated with gene expression levels [3,4,41]. We tested the relationship between the average binding ratios of Nap1 and Htz1 across TSSs and mRNA frequency using previously reported mRNA frequency data [38]. Yeast genes were sorted into five groups according to their mRNA frequency. A negative correlation (*r*<−0.93, *P* value<0.01) between the mRNA frequency and the enrichment of Nap1 and Htz1 surrounding TSSs was observed (Figure 1G). The highly expressed genes exhibited a relative paucity of Htz1 and Nap1 around TSSs (between −200 and +200 bp). In fact, the lack of Htz1 and Nap1 was accompanied by the disassembly of the corresponding nucleosomes (see below). We measured the distance between the two peaks flanking TSSs and discovered that the distance between the two TSS-vicinity peaks for both Htz1 and Nap1 decreased with increasing transcription frequency (*r*≤−0.60) (Supplementary Figure S2). This suggests that Nap1 falls off the chromatin with the loss of Htz1-containing dimers in regions of highly transcribed genes. It is notable that we observed a negative relationship between Htz1 and transcription frequency, as Htz1 incorporation into nucleosomes is a preliminary step in opening chromatin for transcription when the Htz1-containing nucleosome is unstable [5,12]. In yeast, Htz1 is present in promoters as the default state, whereas it is recruited upon activation in mammalian cells. In both cases, however, the presence of Htz1 at the promoter promotes the recruitment of RNAPII [42,43].

### Genome-wide Htz1 distribution in chaperone-deletion mutant strains

To systemically uncover the effects of the Chz1 and Nap1 chaperones on Htz1 deposition in chromatin, we performed protein expression test for Htz1, Htz1 acetylated on K14, and H2A in wild-type, *chz1*Δ, *nap1*Δ and *chz1*Δ*nap1*Δ, deletion strains. We also determined the genome-wide occupancy loci of Htz1 in the deletion strains.

#### Deletions of *chz1* and *nap1* have opposite effect on Htz1 deposition in chromatin

**Figure 2:**
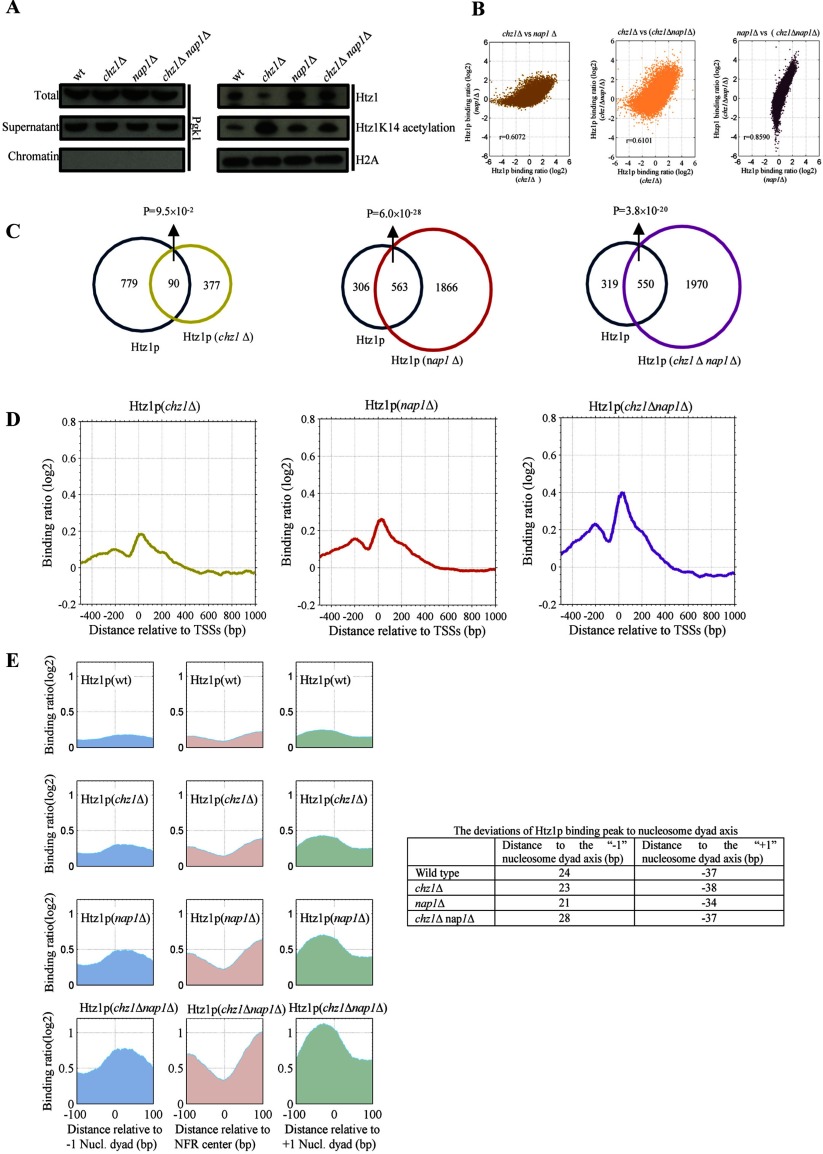
Chz1 and Nap1 have different effects on Htz1 binding to chromatin (**A**) Binding of Htz1 decreases upon deletion of *chz1*, while it increases after deleting either *nap1* or both *chz1* and *nap1*. Acetylation of Htz1 at lysine 14 (Htz1K14ac) increases upon deletion of *chz1*. (**B**) Correlation of Htz1 binding in deletion strains, the correlation coefficient (*r*) is indicated, and the *P* value of Fisher transformation is zero for the three coefficients. (**C**) Venn diagram of Htz1-bound genes in wild-type and gene deletion mutant strains. The Htz1-bound genes are identified by the criteria described in part of Experimental procedures. The hypergeometric distribution *P*-value is indicated. (**D**) The binding patterns of Htz1 around TSSs of 5419 genes in the *chz1*Δ, *nap1*Δ and *chz1*Δ nap*1*Δ strains are shown. The *y*-axis represents the average binding log_2_ ratio. The double-deletion mutant strain shows the highest average binding ratio level, and the *chz1*Δ strain shows the lowest average binding level. (**E**) Htz1 is enriched at the −1 and +1 nucleosomes in deletion mutant strains. The Htz1-binding patterns are shown around the −1 nucleosome dyad, the center of the NFR, and the +1 nucleosome dyad in the wild-type and gene deletion (*chz1*Δ, *nap1*Δ and *chz1*Δ nap*1*Δ) strains. The deviations of the Htz1 binding peak to the nucleosome dyad axis are indicated in the table.

We first carried out a chromatin fractionation assay to compare the levels of Htz1 on chromatin in wild-type and mutant strains (Figure 2A). Surprisingly, Htz1 signals from chromatin were dramatically reduced in the *chz1* mutant. Interestingly, the *nap1* deletion strain had higher Htz1 levels in the chromatin pellet than the wild-type strain. Furthermore, the *chz1–nap1* double mutant also had increased levels of Htz1 on chromatin like the *nap1* single mutant, which indicated that Nap1 may have a dominant role regulating Htz1 incorporation. In contrast, the levels of H2A in chromatin were unaffected in mutant strains.

The result of the chromatin fractionation assay was further supported by ChIP-chip data (Figures 2B, 2C and Supplementary Figure S3). With respect to the Htz1 binding ratio signal, a stronger correlation was observed between the *nap1*Δ and *chz1*Δ *nap1*Δ strains (Fisher transformation, *P* value=0) (Figure 2B). In the *chz1*-deletion strain, only ~1/4 of Htz1-bound genes was the same as in the wild-type strain (Figure 2C). Additionally, 89.6% of the Htz1-bound genes in the wild-type strain lost the Htz1 in the *chz1*-deletion strain (Figure 2C), indicating that Chz1 can distribute Htz1 to specific genes.

In contrast, the number of Htz1-bound genes in the *nap1*-deletion strain was almost three times than that of in the wild-type strain (Figure 2C), suggesting that Nap1 represses the binding of Htz1 to chromatin. Moreover, this effect was enhanced in the double deletion of *chz1* and *nap1* (*chz1*Δ *nap1*Δ) (Figure 2C). More than 63.3% Htz1-bound genes in the wild-type strain were retained in the *nap1*Δ and *chz1*Δ *nap1*Δ strains. In short, deleting *chz1* not only decreased Htz1 deposition in chromatin, but also changed the deposition loci, while deleting *nap1* increased the Htz1 deposition.

To understand whether the association of Nap1 with genomic DNA is dependent on Htz1, we constructed Nap1-myc *htz1* deletion strains. Based on our ChIP-chip data, we chose eight loci with significantly binding with Htz1 and Nap1. We performed the ChIP and RT–PCR to analyse the binding of Nap1 in wild-type and *HTZ1* mutant (Supplementary Figure S11). Interestingly, we discovered that binding of Nap1 at these eight loci is dramatically decreased upon deletion of *HTZ1*, which clearly prove the notion that Nap1-binding is Htz1-dependent.

#### Htz1 occupancy patterns near TSSs in chaperone deletion mutants

We compared the average Htz1-binding ratio profile around TSSs in wild-type and chaperone deletion mutant strains. Regardless of the strain, two peaks flanked a deep trough near the TSS (Figure 2D), showing similarity in the shape of the binding patterns. About 400 bp from the TSS, the intensity of the average binding ratio profile for Htz1 is sorted as *chz1*Δ<wild-type<*nap1*Δ<*chz1*Δ *nap1*Δ, corresponding with the effects of Chz1 and Nap1 on the binding of Htz1 (Figures 2A and 2C). This suggests that the chaperone deletions change the amount of Htz1 in chromatin, but they have little effect on the Htz1 binding pattern shape near TSSs. The genomic coordinates of REG sites (i.e. the DNA regulatory regions, transcription factor binding sites and regulatory variants) were retrieved [37]. We found that Htz1 binding tends to flank REG sites, with the binding profile showing an increase from ±800 bp and a clear valley starting at ~80 bp from the REG sites (Supplementary Figure S4). Htz1 also shows a similar binding pattern near TSSs, which is consistent with previous studies [44,45].

We used the mutant strains to plot the Htz1-binding profiles around the −1 and +1 nucleosomes and the centre of NFRs. The Htz1 occupancy overlaps with the nucleosome occupancy (Figure 2E), suggesting that most of Htz1 located in the chromatin. The binding loci of Htz1 relative to nucleosome dyad sites are also different (*P* value≤4.0×10^−23^) between the −1 and +1 nucleosomes (table on the right of Figure 2E).

### Binding dynamics of Htz1

#### Dynamics of Htz1p binding and Htz1p-bound DNA sequence motifs

Although the average binding ratio profile of Htz1 near TSSs is similar in wild-type and deletion mutant strains, Htz1 binding is decreased at the promoter and increased in the gene body after deleting the chaperones. We divided genomic DNA into three categories: promoter (−500 bp ~ TSS), gene body (TSS ~ TTS) and other region. Deletions of the two chaperones caused an ~9% decrease in number of Htz1-bound probes at promoters (Supplementary Figure S5) providing further evidence that both chaperones play roles in guiding Htz1p to the proper binding sites.

To analyse the regulatory dynamics of Htz1p binding in the deletion mutant strains, 5419 yeast genes were grouped into 40 gene clusters according to their GO annotation (http://www.yeastgenome.org/cgi-bin/GO/goSlimMapper.pl). The average Htz1-binding ratio profile of each cluster was calculated around TSSs. The PCC was calculated using binding profiles for the wild-type and mutant strains, showing the effects of gene deletions on the Htz1-bound genes for the 40 gene clusters.

**Figure 3:**
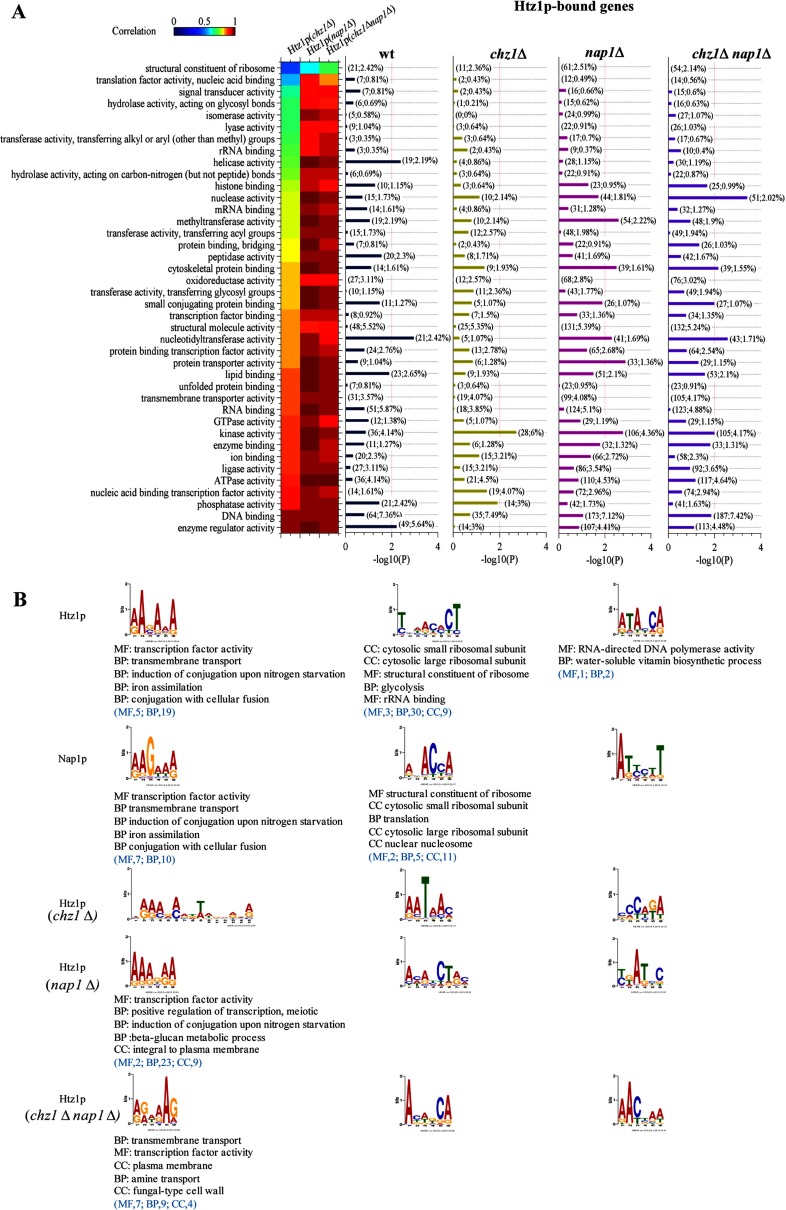
Htz1 preferentially binds purine-rich DNA sequences, and its binding can be changed by Chz1 and Nap1 (**A**) Dynamics of Htz1 binding in wild-type and deletion mutant strains. Yeast genes are classified in 40 clusters based on their functions using GO Slim Mapper (http://www.yeastgenome.org/cgi-bin/GO/goSlimMapper.pl). The heat map indicates the correlation coefficients of the average binding ratio (log_2_) profile (from −500 bp ~TTS) for each gene cluster between wild-type and deletion mutant strains. The bar plots show the *P* value (−log_10_(P)) of the GO enrichment for each function cluster; in the bracket on the bar, the first number indicates the number of Htz1-bound genes of each cluster, and the second number is the percent of the Htz1-bound genes of the cluster relative to the total Htz1-bound genes. The identification of Htz1-bound genes is described in Figure 1(D). (**B**) Motifs of Htz1-bound and Nap1-bound DNA sequences. The DNA sequences of Htz1-bound probes are entered into MEME to generate the binding motif using the default settings. The motif is then inputted into GOMO to find the putative target genes and to analyse their associated GO terms. The summed significant GO terms are listed below the motif if found; the number in the bracket indicates the number of significant terms using the significance threshold *q* value ≤0.05. Abbreviations MF, BP and CC mean molecular function biological process and cellular component, respectively.

Ribosome genes had the lowest Htz1p-binding ratio levels and showed large changes in the binding profile across all deletion mutant strains (Figure 3A and Supplementary Figure S6A). Genes that were associated with nucleic acid binding TFs activity, DNA binding, phosphatase activity and enzyme activity had binding profiles that were highly correlated between the wild-type and mutant strains (Figure 3A). Genes encoding nucleic acid-binding TFs activity showed a typical occupancy profile with Htz1 enriched at the two immediate sides of TSSs regardless of deletion implying that neither Chz1p nor Nap1p affects Htz1 regulation of these genes (Supplementary Figure S6B).

#### Htz1 intrinsically favors AG-rich DNA sequences

We extracted the Htz1-bound genomic DNA sequences and calculated the binding motif of Htz1 using the MEME tool. MEME generated three types of DNA motifs for Htz1 and three for Nap1. The primary Htz1 motif was adenine- and guanine-rich (AG-rich) DNA sequences [Figure 3B (left column) and Supplementary Figure S7]. Importantly, the AG-rich binding feature was not disrupted in the deletion mutants, suggesting that Htz1 intrinsically favours AG-rich DNA sequences. The primary Nap1 motif was very similar to that of Htz1, as would be expected by the findings that Htz1 and Nap1 bind the same genomic loci in wild-type and supporting the hypothesis that Nap1 competitively binds Htz1-contianing dimers.

We used GOMO to track the target genes of the motifs. Results indicated that the target genes of the AG-rich motif associates with transcription factor activity and transmembrane transport process [Figure 3B (left column)]. The secondary Htz1 motif is associated with ribosomal subunit constituents [Figure 3B (middle column)]. The second tertiary motifs are completely disrupted in the deletion mutants. This is more obvious in *chz1* mutants suggesting that Chz1 partly contributes to the Htz1-binding specificity.

Htz1 favours TATA-less promoters [3]. Our results showed that the average Htz1-binding ratio profile for TATA-less genes was higher than that for TATA box-containing genes in both wild-type and deletion mutants (Supplementary Figure S8). Because TATA box-containing genes are highly regulated and TATA-less genes perform more housekeeping functions [39], which generally have high expression levels, we inferred that Htz1 enrichment at TATA-less promoters caused nucleosome instability and facilitated the disassembly of nucleosomes near TSSs during transcription initiation, which is consistent with previous study [3].

### Transcriptome analysis

#### Nap1 is dominant to Chz1 in gene expression regulation

**Figure 4:**
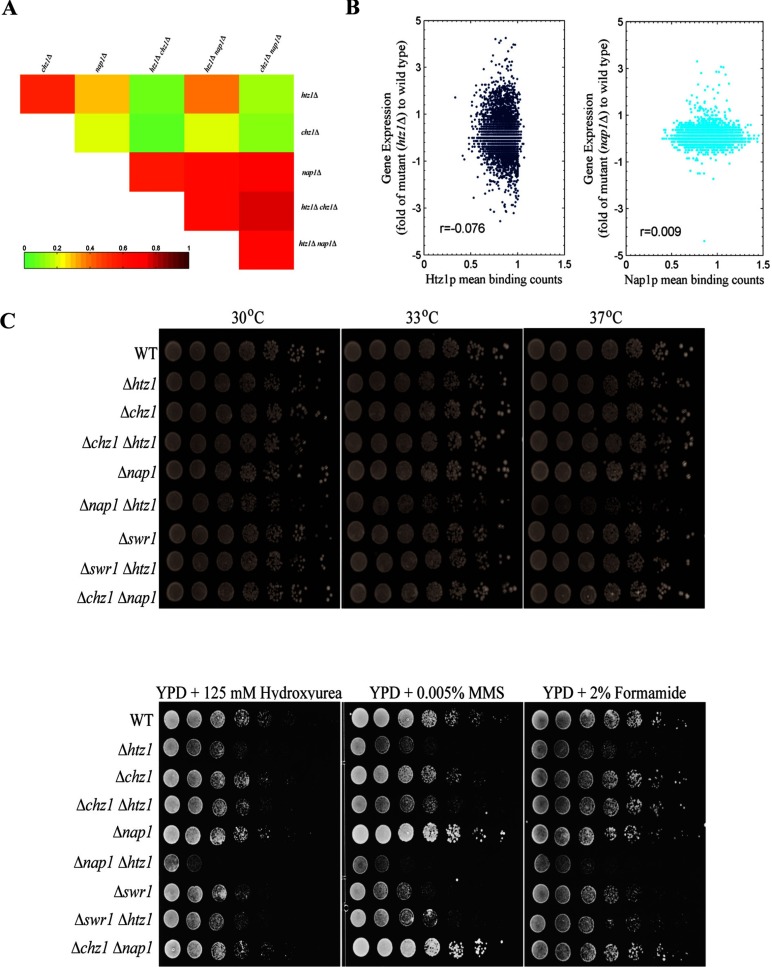
Nap1 function is dominant in gene expression (**A**) Correlation heat map of gene expression between the *htz1*Δ, *chz1*Δ, *nap1*Δ, *htz1*Δ *chz1*Δ, *chz1*Δ *nap1*Δ and *htz1*Δ *nap1*Δ strains. Gene expression is represented as the expression level fold change between mutant and wild-type strains. The correlation coefficient is calculated for 5419 genes. (**B**) A plot of gene expression versus Htz1 mean binding. The Htz1 mean binding is the average binding ratio in the promoter region (−500–0 bp relative to the TSS); the gene expression is the fold change between the mutant and wild-type strains. (**C**) Growth of yeast with deletions of *chz1*, *nap1*, *htz1* and their combinations.

Although the histone chaperones were proposed to be involved in the incorporation of histone variants into chromatin, we wanted to understand whether histone chaperones could regulate the transcriptome. We performed genome-wide transcriptome analysis of the *htz1*Δ, *nap1*Δ, *chz1*Δ *nap1*Δ, *chz1*Δ *htz1*Δ and *nap1*Δ *htz1*Δ strains. The correlation analysis showed that the transcriptome profile of *htz1* was poorly correlated with that of both *chz1* and *nap1* (Figure 4A). These data showed that histone chaperones may regulate transcription and this role is independent of the association with the histone variant Htz1. Interestingly, Nap1 showed strong dominant roles in regulating transcription. The transcriptome profile of the *nap1 chz1* double-mutant strain strongly correlated with that of the *nap1* single mutant, which supported the dominant role of Nap1 in regulating the incorporation of the histone variant Htz1 into chromatin.

#### No significant connection between Htz1 and Nap1 binding and the corresponding transcripts

Because both *htz1* and *nap1* deletion can lead to dramatic global changes in the transcription of many genes, we wanted to understand whether there was a direct link between the binding of these proteins and the transcriptional changes that followed deletions. We observed that there was no statistically significant connection between Htz1 and Nap1 binding and the corresponding transcripts (Figure 4B).

#### *chz1* and *nap1* do not genetically interact, Htz1 and Nap1 have partial functional redundancy

We assayed genetic interactions between Htz1 and histone chaperones Chz1 and Nap1. As shown in Figure 4(C), *chz1* and *nap1* did not show any genetic interaction. However, the *nap1 htz1* double mutant showed a growth defect upon a shift to 37°C, suggesting some functional redundancy between Htz1 and Nap1. We also tested whether the genetic interaction between Htz1 and histone chaperones will exist on different drug-containing plates. As showed in Figure 4(C), *htz1* single mutant was very sensitive to the presence of the DNA-damaging agents methyl methanesulfonate, hydroxyurea and formamide, which indicated that Htz1 may have an important role in genome stability. However, the *chz1* and *nap1* single mutants did not show a growth defect on these plates. Consistent with previous findings that *nap1* genetically interacts with *htz1*, *htz1 nap1* double mutants grew even slower than the *htz1* single mutant on these drug-containing plates. As previously reported, functional role of Htz1 is partially involved in the loss of Swr1 activity. We also tested the sensitivity of the *swr1* mutant on these plates. Although the *swr1* mutant also showed sensitivity to DNA damaging agents, *htz1* mutant grew slower than the *swr1* mutant, which indicated that Htz1 may have Swr1-independent functions to regulate genome stability.

### Acetylation of Htz1

#### Deletion of *chz1* increases the acetylation of Htz1

**Figure 5:**
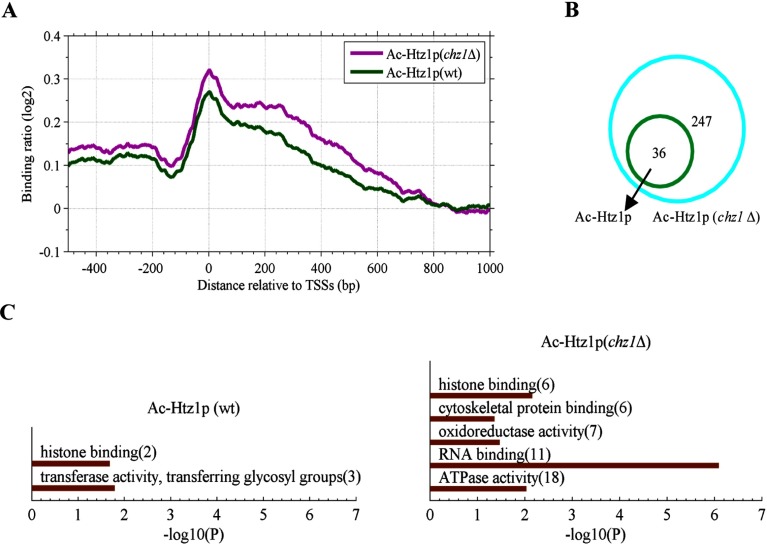
Deletion of *chz1* increases Htz1K14ac (**A**) Average binding ratio profiles of Htz1K14ac in the wild-type and *chz1*Δ strains. (**B**) A Venn diagram shows the number of acetylated Htz1-bound genes in the wild-type and *chz1*Δ strains. (**C**) Gene ontology analysis for acetylated Htz1-bound genes in the wild-type and *chz1*Δ strains.

Interestingly, deleting *chz1* increased the acetylation level of Htz1 (ac-Htz1). Around the TSSs, the average binding profile of ac-Htz1 in the *chz1*Δ mutant was obviously higher than that of the wild-type strain (Figure 5A). Furthermore, the number of ac-Htz1-bound genes of the *chz1*Δ mutant was also greater than that of the wild-type strain (Figure 5B). GO analysis indicated that histone binding-related and RNA binding-related genes were significantly associated with ac-Htz1 when *chz1* was deleted (Figure 5C). Perhaps this acetylation reflects a compensatory mechanism to silence the effects of additional Htz1 incorporation.

### Nucleosome-positioning dynamics

#### Nucleosome occupancy of Htz1-bound genes decreases with *htz1* or *chz1* deletion but does not change with *nap1* deletion

We determined the genome-wide nucleosome positions by next generation sequencing. The nucleosome occupancy near TSSs of 5419 yeast genes is shown in Supplementary Figures S9 (A)–(D). The average read intensity near TSSs is shown in supplementary Figure S9(E). The typical distribution pattern was repeated in both the wild-type and mutant strains (Figure S9E). An NFR is shown around TSSs and REG sites (Figures S9E and S10). The NFR was flanked by two well-positioned nucleosomes and a phased organization of nucleosomes (nucleosome array) was observed downstream of the TSS.

**Figure 6:**
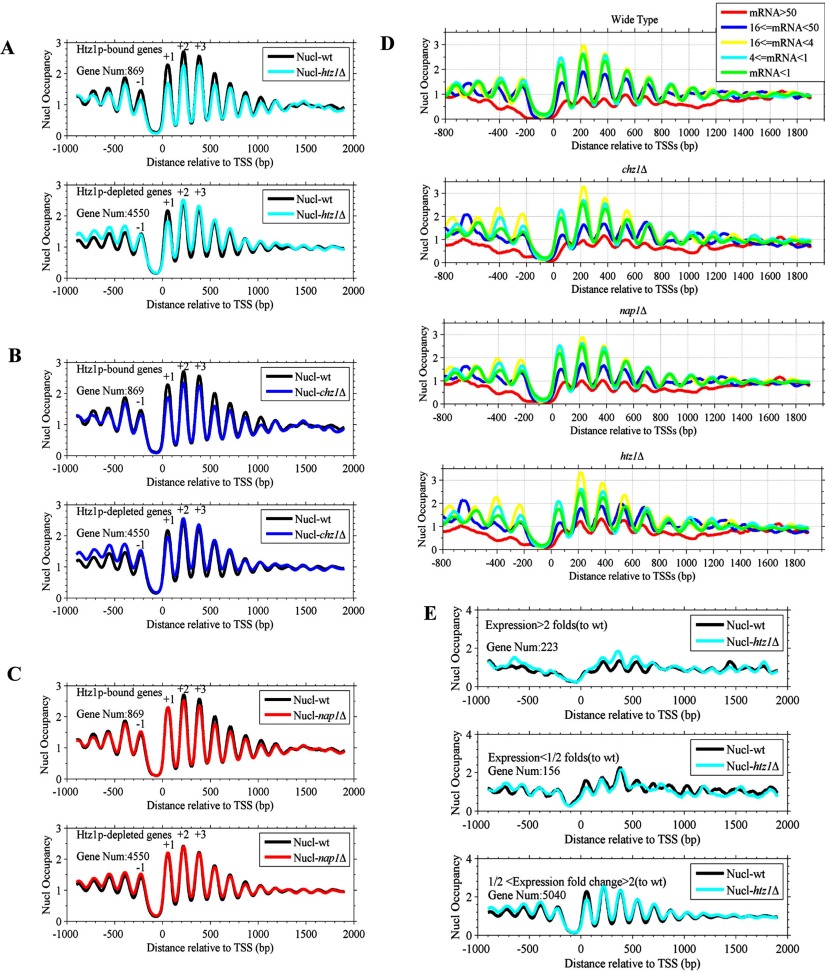
The average nucleosome occupancy of Htz1-bound genes changes with gene deletion and transcription frequency (**A**–**C**) Nucleosome occupancy profiles for Htz1-bound genes in wild-type, *htz1*Δ, *chz1*Δ and *nap1*Δ strains. The Htz1-bound genes are identified in the wild-type strain. (**D**) Nucleosome occupancy profiles for genes with different transcription frequency (transcripts/h). (**E**) Nucleosome occupancy profiles for genes with differential expression (expression>2-fold; expression<1/2-fold). The fold change of expression is measured between the wild-type and *htz1*Δ strains.

We identified 869 Htz1-bound genes in the wild-type strain. Although the gross levels of the average nucleosome occupancy near TSSs were not significantly different (Figure S9E), the nucleosome occupancy of Htz1-bound genes dramatically decreased with *htz1* deletion, especially for the −2, −1, +1 and +2 nucleosomes near TSSs (Figure 6A). In contrast, the nucleosome occupancy of non-Htz1-bound genes (Figure 6A) did not significantly change in the wild-type and *htz1*Δ mutant strains except at the +1 nucleosome (Figure 6A). Moreover, we also found that Htz1-bound genes had lower nucleosome occupancy in the *chz1*Δ mutant than in the wild-type strain (Figure 6B). These results indicate that H2A cannot compensate for Htz1 deficiency to assemble nucleosomes at Htz1-bound genes.

Unlike the *chz1*Δ or *htz1*Δ mutants, the nucleosome occupancy in the *nap1*Δ mutant was not different from that in the wild-type strain (Figure 6C). This indicated that deleting *htz1* or *chz1* disassembled nucleosomes near the TSS of Htz1-bound genes, whereas deleting *nap1* did not have an effect on the nucleosome occupancy of Htz1-bound genes. This can be explained by the effects of Chz1 and Nap1 on Htz1 deposition. There are Htz1-containing nucleosomes near the TSS of Htz1-bound genes. Deleting *htz1* results in the disassembly of the Htz1-containing nucleosomes, so we observed the decreased nucleosome occupancy. Deleting *chz1* can also decrease Htz1 in chromatin, which decreases the nucleosome occupancy. Deleting *nap1* increases Htz1 deposition in chromatin. That is, Htz1 in chromatin is sufficient to form Htz1-containing nucleosomes. Thus, the nucleosome occupancy is not different between the wild-type and the *nap1*Δ mutant strain.

#### A broader NFR is associated with highly transcribed genes

We plotted the nucleosome occupancy profiles for the genes with different transcription frequency (Figure 6D). A broad NFR and a low nucleosome occupancy level are associated with highly transcribed genes in both the wild-type and mutant strains. This is consistent with previous reports [46,47]. We also noticed that the genes with a great change in expression (>2-fold or <1/2-fold compared with wild-type strain) were associated with a broad NFR (Figure 6E).

## DISCUSSION

Incorporating Htz1 into chromatin is required for transcription, DNA replication, DNA repair and chromatin integrity [2,4]. The process involves a chaperone network [29]. Both Nap1 and Chz1 are involved in the deposition of Htz1 in chromatin [18,23,26]. We revealed the distinct roles of the two chaperones in escorting Htz1 from the cytoplasm to the nucleus and to chromatin.

First, according to previous reports [26,30] and our results, Nap1 is the Htz1 chaperone both in the cytoplasm and in chromatin: it is involved both in importing Htz1 from the cytoplasm to the nucleus and in incorporating Htz1 into nucleosomes. Moreover, Nap1 controls nucleosome quality by eliminating non-nucleosome histone–DNA interactions [26,48]. Also, Nap1 is involved in both nucleosome assembly and disassembly [29,48–50]. In contrast, there is no evidence that Chz1 is associated with Htz1 in the cytoplasm [30]. Our finding suggested that Chz1 does not directly interact with chromatin. Htz1–H2B dimers only interact with Chz1 in the nucleus [18,30]. Secondly, our results suggested that Chz1 facilitated Htz1 deposition in chromatin although Chz1 does not physically interact with genomic DNA. We observed increased Htz1 levels in chromatin in the *nap1*Δ strain, suggesting that Nap1 represses Htz1 deposition. This is partly due to two mechanisms: Nap1 can competitively bind to DNA with non-nucleosome histone–DNA interactions [26,48], and Nap1 can disassemble nucleosomes [29,48–50]. Nap1 is dominant to Chz1 in Htz1 deposition, *chz1*Δ*nap1*Δ having nearly identical phenotypes with *nap1*Δ. We also discovered that Nap1 and Chz1 appear to have little impact on nucleosome positioning because we only observed changes in nucleosome occupancy and not in nucleosome position. Nucleosome dynamics are probably mediated by chromatin remodelling machines [49]. We found that the −2, −1, +1 and +2 nucleosomes contain more Htz1 than other nucleosomes. Moreover, the Htz1-containing nucleosomes disassemble if the Htz1 supply is reduced (*htz1*Δ or *chz1*Δ). This suggests that Htz1 deposition is specific for those Htz1-bound genes. That is, H2A cannot completely compensate for Htz1 deficiency at Htz1-bound genes. Htz1-bound DNA sequences are adenine- and guanine-enriched. Finally, we observed that the association between the transcription frequency and nucleosome occupancy near TSSs was not changed in the deletion mutants.

Deposition of Htz1 on promoters has been showed to be typical yeast genes whose transcription is frequently reprogrammed [51]. Two Htz1-containing nucleosomes flank the NFR, serving to poise quiescent genes for activation and transcriptional initiation [52]. No correlation between Htz1 binding and changes in gene expression in HTZ1 mutant, and Htz1 prefers to associate with low-frequency transcription genes further demonstrated the binding of Htz1 appears to establish an epigenetic markers for the rapid activation of its associated promoters [35]. Thus, we thought that Htz1 deposition at promoters is a preparation for transcription. The triggering of transcription needs other events, such as TF and Pol II binding. That is, alteration of Htz1 binding will change the transcription preparation for quiescent genes. Similar to HTZ1 mutant, there is no significant correlation between Nap1 binding and deregulated transcription in NAP1 mutant. Histone chaperone Nap1 has been implicated to eliminate the non-nucleosomal histone DNA interaction [26]. It will be quite interesting to fully understand the functional significance of Nap1 binding. Additionally, as shown in Figure 3(A), kinase activity genes are enriched for Htz1 binding when the chaperone was deleted. However, helicase activity genes and enzyme regulator activity genes were no longer enriched for Htz1 binding in the deletion mutant strains, probably due to the overall increase in binding, whereas phosphatase activity genes were enriched only in the chz1 deletion mutant strain, probably due to decreased overall binding.

According to literatures and our present work, we proposed a model to illustrate the mechanisms of Nap1 and Chz1 in depositing Htz1 in yeast (Supplementary Figure S12). In this model, Nap1 and Chz1 have different roles in Htz1 deposition. Nap1 binds with Htz1–H2B dimer, forming a Nap1–Htz1–H2B complex, in cytoplasm and then the complex is imported into the nucleus. In nucleus, Htz1–H2B–Nap1 interacts with SWR1 and completes the deposition of Htz1. Chz1 does not associate with Htz1 in cytoplasm. Htz1 deposition mediated by Chz1 is occurs in nucleus. During the nucleosome disassembly under conditions of transcription, DNA repair and other process, Htz1–H2B is released from chromatin into the nucleus. For the deciduous Htz1–H2B, their deposition need help of Chz1. Chz1 catches the released Htz1–H2B and delivers it to SWRI and realize the deposition. However, there is another possibility to explain the increased level of Htz1 incorporation in the absence of Nap1. Rather than Nap1 and Htz1 competing for binding to the same genomic loci, it may be likely that the absence of Nap1 may also decrease the incorporation of conical H2A, which would allow Htz1/H2B dimmers to assemble in inappropriate places.
